# Dr. Hayd Replies to J. P. and J. H.

**Published:** 1890-11

**Authors:** H. E. Hayd

**Affiliations:** 78 Niagara Street


### DR. HAYD REPLIES TO J. P. AND J. H.

*Editors Buffalo Medical and Surgical Journal:*

I AM very pleased that J. P. and J. H. considered my letters of sufficient interest to answer them through the columns of your journal.

Yes, "a follower of the prophet" has returned to Buffalo, with his faith strengthened in the "teachings of his master," and better acquainted with the tenets of electro-therapeutics. Moreover, "he wandered to Mecca," as he felt that there was much for him to learn in this question of electricity — a subject which is engaging the attention and interest of many of the best men in the profession.

I object, when my learned correspondents say that surgeons are foremost in narrowing the usefulness of electricity, in view of the fact that many of the best operators of to-day are among the most earnest advocates of its employment. I need but refer to Keith, Sr., who says that all bloody operations for the relief of fibroids should be abandoned until electricity is first tried, so satisfactory and encouraging were the results in his hands. Sir Spencer Wells, and Playfair, endorse it in equally commendatory terms, as well as Savage, Noeggerath, Engelmann, Thomas, Mundé, Zweifel, La Torre, Delétang, Mann, Grandin, and many others. It seems to me that these men may lay just claim to the confidence of J. P. and J. H., and might be looked upon by them as "authorities in the diagnosis, pathology, and treatment of most of the conditions" which come under the care of the ordinary gynecologist. Whether Apostoli now advocates "electro-puncture in extra-uterine pregnancy," — if he ever did, my authority being J. P. and J. H.,— does not lessen his ability as a diagnostician today. It seems to me that a well-educated man, who has enjoyed the privileges of a large clinic for a great many years, having treated over three thousand patients at his public dispensary alone, together with the experience derived from a large private practice, ought to be in a position to make a diagnosis satisfactory to even J. P. and J. H. His clinic has been honored by visits from England and Germany's most representative men, as well as many persons of position and reputation in our own country. These men return to their homes satisfied with his results, and use electricity in their own practices with satisfaction to themselves. That "these men create a disease, and then cure it, and juggle with pathological terms to mask a physiological variation," is absurd on the face of it. Perhaps J. P. and J. H. are men quite familiar with this juggling process, and use high-sounding platitudes and beautiful rhetoric to picture their own imaginations.

Yes, a great many fibroids are seen, and I think I can say in all fairness, more than one sees in any other clinic of the same size in Europe. This claim is not unreasonable, when we take into consideration that Dr. Apostoli was among the first to advocate electricity in the treatment of these growths. His clinic is situated in a large city, and has been supplied for years by the material of many interested confréres. True, many cases of fibroid require no treatment, and I have seen such cases, where no special symptoms were complained of, dismissed without treatment, and invited to return to the hospital when assistance might be required. The cases which I referred to in my letters, were those in which treatment was necessary to relieve the severe pain, dangerous and exhausting hemorrhages, and other pressure symptoms, which were complained of. These patients have been relieved, and, to all intent and purpose, cured, since they have required no treatment for a great many years, and have been enabled to do their work and enjoy themselves. They have been kept under observation, in that they present themselves at variable intervals at the clinic, in order that their condition may be noted. That J. P. and J. H. found anything in this to criticise, is beyond my power to explain or understand. These observations have been verified by other men, who, in their practices, have relieved the symptoms and have seen the tumors appreciably diminish in size and sometimes disappear under treatment.

I should have been particularly interested if J. P. and J. H. had given us their experiences with electricity, and had not in a general way abused the agent and the men who find it of service in their practice. Is not the reputation of these men whom I have named, and many others I shall be pleased to give, sufficiently honorable, and their practices sufficiently remunerative, to protect them from the charge of belonging to "that class who resort to Louisiana lottery schemes to influence their patients and gain their confidence and respect?" Perhaps J. P. and J. H. have been buoyed up in the belief that by throwing mud, in the shape of mean insinuation and petty slander, some might stick and thus make their argument more forcible. Let me admonish them that that kind of practice will not settle these points, upon which the profession is in serious study. The wonderful ability which they possess, as evidenced by their readiness in criticism, might be better spent in working up some of these difficult questions that Dr. Apostoli is laboring so assiduously to explain. Surely he better invite the collaboration of J. P. and J. H., as the profession is losing too much in having so much of their energy misdirected and wasted.

No more claim for accurate diagnosis is made for the Rue de Jour than would be for any other clinic in Europe, conducted by able and educated men. Moreover, no more brilliant diagnoses are made than would be made by capable and experienced men elsewhere. Mistakes occur, as they do in every other hospital — and perhaps even in the practice of J. P. and J. H.— and are as willingly admitted by Apostoli as they would be by any other reasonable and sensible man. Moreover, these diagnoses are made *coram publico*, and distinguished men present are invited to express an opinion, and thus be fully satisfied with the subsequent treatment of the cases. I don't think that it is necessary for J. P. and J. H. to exercise any anxiety that the readers of the BUFFALO MEDICAL JOURNAL will be unduly carried away by the "wild enthusiasm" of the correspondent who wrote two letters from Paris. Whatever that correspondent's utterances may have been in April last, they were influenced by an honesty of purpose, and a personal experience of Apostoli's methods — which J. P. and J. H. question — as laid down in the more recent works and publications on electricity, and which he made himself thoroughly familiar with before commencing the use of this agent in his practice. Those remarks were further prompted by an experience of fifteen months, in a reasonably large number of cases, in which careful records were kept, and by results more satisfactory than he obtained by any of the other methods familiar to him, as laid down in recognized works on diseases of women, and the teachings of able men whom he honestly, faithfully, and studiously followed. Yes, Bigelow, Massey, Liebig and Rohé, Erb, and others, tell him, as they do J. P. and J. H., what they can expect to accomplish with electricity, and the diseases cured by it when properly employed. Nor need they war with themselves and their supposed fancies about electro-puncture, but, on the contrary, try this "simple prick," which Apostoli, Mundé, Goelet, and others, have found a sovereign remedy, before they offer any opinion as to its utility.

I would also suggest that they consult Thomas, Skene, Athill, and many other recognized and recent works on diseases of women, and they will find that the term "cellulitis" is applied to that form of inflammation involving the cellular tissue in the broad ligaments and about the supra-vaginal portion of the cervix uteri (Skene — page 556). I used the term as it is most familiar to English-speaking people — in fact, used in preference in Soho Square and other large special hospitals in London — and so willingly accepted by the most recent authors that they commence the discussion of this form of inflammation under the title "pelvic cellulitis." They know, Mr. Editor, and so did your Paris correspondent, that an inflammation does not confine itself purely and simply to one class of tissue, but usually involves, to a greater or less extent, adjacent structures as well. Authors are satisfied with the term because it is practical, convenient and expressive, although not thoroughly scientific. Recent works in pathology are not necessary to inform the readers of the BUFFALO MEDICAL AND SURGICAL JOURNAL on this question, nor was the brilliant suggestion of J. P. and J. H. I wonder if they are juggling with terms to appear learned and transcendental, since brilliant, educated and scientific men continue to be satisfied with an old nomenclature?

The pain, as I said in my letter, is not great, if care be exercised in the use of the external pad — at all events, not sufficient to be a barrier to the employment of this powerful agent.

Hemorrhage is checked, no matter what J. P. and J. H. say to the contrary, and, I believe, more permanently than by any other method that the general profession is acquainted with. That it fails in some cases, may be true, but on this account must it be relegated to disuse, and considered worthless and unreliable? If they read my letters more carefully, they will see that I said the patient bled profusely, after an examination, and that sixty ma. positive galvanism completely checked the hemorrhage, the séance lasting about ten minutes.

True, some cases of menorrhagia and metrorrhagia seem to be aggravated by electrical treatment, and I suggested that possibly too small carbon tips were used, and that the mucous membrane of the uterus was not thoroughly cauterized over its entire surface. In this opinion, Dr. Goelet bears me out by his expressions as published in the *Archives of Pediatrics and Gynecology*, August, 1890.

Perhaps J. P. and J. H. will find more to convince and less to discourage them in electricity, if they learn how to use it properly, and follow out the details of treatment more carefully, and I would suggest a visit to Apostoli's clinic to further their knowledge in this important branch of therapeutics.

H. E. HAYD.

78 NIAGARA STREET, Oct. 10, 1890.

---

### THE HOSPITAL GRADUATE DEGREE.

*Editors Buffalo Medical and Surgical Journal:*

